# Pregnancy Followed by Delivery May Affect Circulating Soluble Lectin-Like Oxidized Low-Density Lipoprotein Receptor-1 Levels in Women of Reproductive Age

**DOI:** 10.1155/2012/837375

**Published:** 2012-04-26

**Authors:** Mehmet Balin, Ahmet Çelik, M. Ali Kobat, Adil Baydas

**Affiliations:** ^1^Department of Cardiology, Elazig Education and Research Hospital, Elazig 23100, Turkey; ^2^Department of Cardiology, Mus Government Hospital, Mus 49100, Turkey

## Abstract

*Background/Objective*. It is known that menopause or lack of endogenous estrogen is a risk factor for endothelial dysfunction and CAD. Lectin-like oxidized low-density lipoprotein receptor-1 (LOX-1) is involved inmultiple phases of vascular dysfunction.The purpose of the current study was to determine the association between soluble LOX-1 (sLOX-1) and pregnancy followed by delivery in women of reproductive age. *Materials/Methods*. Sixty-eight subjects with pregnancy followed by delivery (group 1) and 57 subjects with nongravidity (group 2) were included in this study. Levels of sLOX-1 were measured in serum by EL SA. *Results*. Plasma levels of sLOX-1 were significantly lower in Group 1 than Group 2 in women of reproductive age (0.52 ± 0.18 ng/mL and 0.78 ± 0.13, resp., *P* < 0.001). There were strong correlations between sLOX-1 levels and the number of gravida (*r* = −0.645, *P* < 0.001). The levels of sLOX-1 highly correlated with the number of parous (*r* = −0.683, *P* < 0.001). * Conclusion*. Our study demonstrated that serum sLOX-1 levels were associated with pregnancy followed by delivery that might predict endothelial dysfunction. We conclude that pregnancy followed by delivery may delay the beginning and progress of arteriosclerosis and its clinical manifestations in women of reproductive age.

## 1. Introduction

Even though substantial efforts have been made to improve education and public awareness and despite the use of effective medications and life-style changes for controlling the associated risk factors, coronary artery disease (CAD) remains the leading cause of death in women worldwide [1, 2]. In contrast to age-matched men, the incidence of clinical manifestations of CAD is considerably lower in premenopausal women; however, most of women develop CAD after menopause when endogenous estrogen levels are low [3–5].

During normal menstrual cycles, women show high levels of estrogen just before ovulation and during the luteal phase and in the normal physiology of pregnancy, women have significantly higher levels of estrogen derived mainly from the placenta [6]. Estrogens have been known to exert various positive effects on the cardiovascular system [7, 8]. It has thus been shown that estrogens retard the atherosclerotic process and induce rapid vasodilatation through the production of an endothelium-derived vasoactive mediator, nitric oxide (NO) [8–10]. Hashimoto et al. [11] reported that endothelium-dependent vasodilatation is increased in young women during the phases of their menstrual cycles when endogenous estrogen levels are high, and pregnant women show significantly high levels of estrogen. Some studies have documented that estrogens are potent antioxidants and decrease low-density lipoprotein cholesterol (LDL-C) oxidation in vitro and in vivo [12, 13]. While estrogens decrease lipid peroxidation and formation of reactive oxygen species [14], androgens and progestins increase oxidative stress parameters [15]. 

Lectin-like oxidized low-density lipoprotein receptor-1 (LOX-1), a type II membrane glycoprotein, is the major receptor for oxidized low-density lipoprotein (ox-LDL) in endothelial cells [16]. Oxidative stress and ox-LDL both alter endothelial biology by activating a specific receptor LOX-1. The activation of LOX-1 has been shown to lead to further oxidative stress in endothelial cells and the appearance of proinflammatory phenotype [17]. LOX-1, furthermore, is cleaved at the membrane-proximal extracellular domain by proteases [18, 19] that may also be associated with endothelial dysfunction and atherosclerotic plaque formation and destabilization, resulting in soluble LOX-1 (sLOX-1) release into the circulation [19]. Since the level of soluble receptors in circulating blood may reflect the expression of membrane proteins and disease activities, sLOX-1 may be a potential biomarker of vascular disease assessment. 

Therefore, we hypothesized that if women have been exposed for a longer time and/or at a higher level to endogenous (not exogenous) estrogen, such as pregnancy followed by delivery and/or gravidity, they may obtain estrogen's beneficial “cardioprotective,” “antiatherosclerosis,” and/or “antioxidant” effect. The purpose of the current study was to determine the association between pregnancy followed by delivery and sLOX-1.

## 2. Methods

### 2.1. Patient Population

From January 2010 to June 2011, we prospectively evaluated 1284 patients in cardiology outpatient clinic of our hospital. Sixty-eight subjects with pregnancy followed by delivery (Group 1) and 57 subjects with nongravidity (Group 2) were included in this study. All participants provided written informed consent to participate in the study. The protocol was approved by the local Ethics Committee.

Exclusion criteria included pregnancy, known polycystic ovary syndrome, congestive heart failure (ejection fraction <50%), myocardial infarction, stroke, known peripheral atherosclerotic disease, surgical coronary intervention, other major vascular surgical procedures, coronary angioplasty, unstable angina pectoris, diabetes mellitus, hypertension, suspected myocarditis or pericarditis, impaired renal function (creatinine ≥1.4 mg/dL), unstable endocrine or metabolic diseases known to influence serum inflammation markers, concomitant inflammatory diseases such as infections and autoimmune disorders, active or chronic hepatic/hepatobiliary disease, and malignancy. Patients taking oral contraceptive, corticosteroids, anti-oxidant vitamins, and alcohol were also excluded from the study.

### 2.2. Blood Sampling and Laboratory Methods

Blood samples of all individuals were taken from an antecubital vein following an overnight fasting state at the first three days of menarche. After centrifugation at 3000 ×g for 10 minutes, serum and plasma samples were frozen and stored at −80°C until an assay could be performed. Serum sLOX-1 levels were measured by a commercially available enzyme-linked immunosorbent assay kit (USCN Life Science, Wuhan, China). The detection limit for serum sLOX-1 level was 2.4 pg/mL with a coefficient of variation <5%. Triglyceride (TG), total cholesterol (tot-C), LDL-C, and high-density lipoprotein cholesterol (HDL-C) concentrations were measured by automated chemistry analyzer (Roche Diagnostics, Indianapolis, USA) by using commercially available kits.

## 3. Statistical Analysis

Continuous variables were given as mean ± SD; categorical variables were defined as percentages. Comparisons between Group-1 and Group-2 were carried out using an independent samples *t*-test. Correlation analyses were performed using the Pearson coefficient of correlation. SPSS 15.0 software was used for basic statistical analysis (Version 15, SPSS Inc., and Chicago, IL, USA). A value of *P* < 0.05 was accepted as statistically significant.

## 4. Result

The clinical and demographic characteristics of study subjects were summarized in Table 1. The mean age was 33.5 ± 6.1 years in pregnancy followed by delivery group and 35.5 ± 7.5 years in nongravidity group (*P* = 0.1). The mean age of first menarche was 12.1 ± 2.3 years in pregnancy followed by delivery group and 11.9 ± 1.9 years in nongravidity group (*P* = 0.5). The rates of family history and smoke were similar between the two groups (Table 1). The smoker subjects in Group 1 had 3.5 ± 1.3 pack-year history of smoking, and smoker subjects in Group 2 had 5.0 ± 2.1 pack-year history of smoking (*P* = 0.1). The levels of total-C, LDL-C, HDL-C, and triglyceride were also similar between the two groups (Table 1).

**Figure 1:**
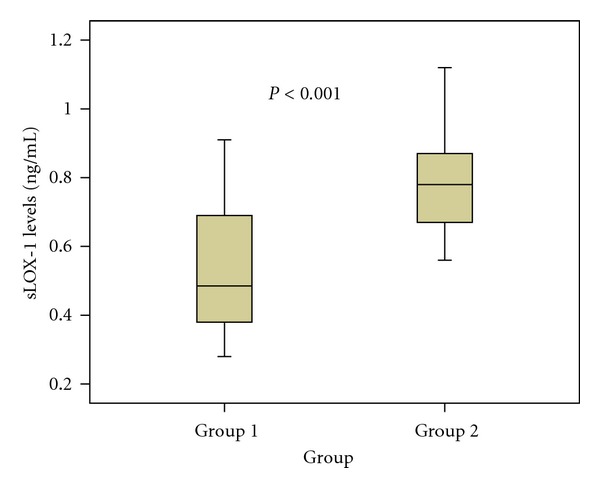
The comparison of sLOX-1 levels between two groups (Group 1: pregnancy followed by delivery group, Group 2: nongravidity group. Soluble LOX-1 levels were 0.78 ± 0.13 ng/mL in nongravidity group and 0.52 ± 0.18 ng/mL in pregnancy followed by delivery group, *P* < 0.001).

**Figure 2:**
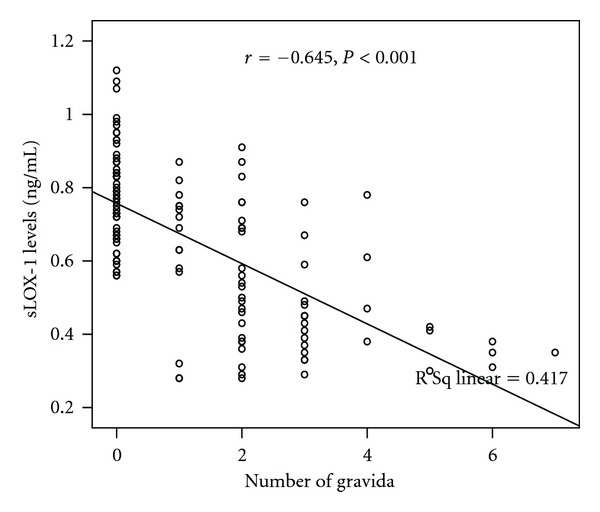
The correlation between sLOX-1 levels and number of gravida.

**Figure 3:**
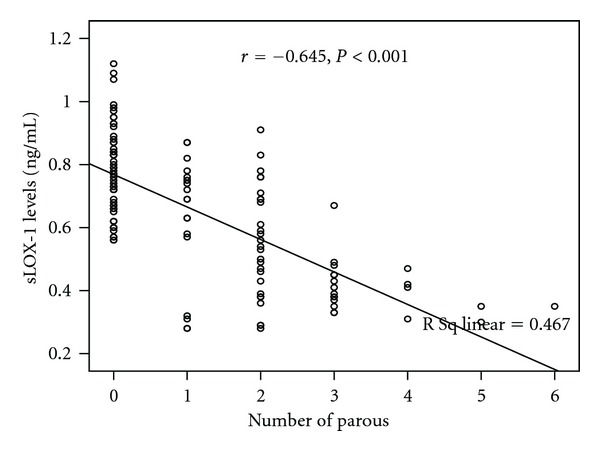
The correlation between sLOX-1 levels and number of parous.

Figure 1 shows the sLOX-1 levels between two groups. The sLOX-1 levels were significantly higher in nongravidity group than pregnancy followed by delivery group (0.78 ± 0.13 ng/mL and 0.52 ± 0.18 ng/mL, resp., *P* < 0.001). The sLOX-1 levels highly negatively correlated with the number of gravida (Figure 2, *r* = −0.645, *P* < 0.001).  Figure 3 shows a highly negative correlation between sLOX-1 levels and number of parous (*r* = −0.683, *P* < 0.001). The sLOX-1 levels were not correlated with age and age of first menarche (*r* = 0.055, *P* = 0.541 and *r* = −0.015, *P* = 0.865, resp.). In the multiple linear regression analysis age was positively related and number of gravity was negatively related with sLOX-1 levels (for age *P* = 0.011, beta = 0.169, *t* = 2,589 for parous *P* < 0.001, beta = −0.713, *t* = −10,912).

## 5. Discussion

To the best of our knowledge, this is the first study that shows the relationship between sLOX-1 levels and pregnancy followed by delivery in women of reproductive age. This study showed that women who had at least 1 pregnancy followed by delivery showed a decreased level of sLOX-1 compared with those who had never experienced delivery. The sLOX-1 levels had significantly negative correlation with gravida and parous. We hypothesized that if women have been exposed for a longer time and/or at a higher level to endogenous (not exogenous) estrogen, such as pregnancy followed by delivery and/or gravidity, they may obtain estrogen's beneficial effect and may have a greater decrease in level of sLOX-1. These findings may support the idea that as long as women are exposed to endogenous estrogen they have decreased level of sLOX-1.

Coronary artery disease remains the leading cause of death in the 21st century. Despite the advances in this area, it is still the main cause of death among women in developed countries [20]. The prevalence of CAD in premenopausal women is smaller than in postmenopausal women, when there is an exponential increase, making the risk for women equal to that for men by the age of 65–70 years. This lag concerning the age period at which the frequency of cardiovascular events increases among women as compared to men has been ascribed to the actions of endogenous estrogen on the cardiovascular system, through mechanisms as yet not completely clarified.

The well-known risks for CAD, such as systemic hypertension, smoking, obesity, sedentary life-style, dyslipidemia, stress, family history of CAD, diabetes mellitus, menopause, lack of endogenous estrogen, and insulin resistance, are numerous [21]. More recently, endothelial vascular dysfunction has become suspected as being associated with CAD. The term endothelial dysfunction is more frequently used to refering reduction in endothelium-dependent vasodilatation, associated with diminished bioactivity of local vasodilative factors (especially NO). Data from prospective trials have been confirming the hypothesis that endothelial dysfunction precedes the emergence of chronic disorders. Currently, it is a consensus that endothelial dysfunction is the initial event in development of atherosclerosis [22]. There are many techniques for investigating the endothelial function, from those that focus on cellular and molecular aspects, through methods involving tissue culture and molecular biology tools, to clinical trials applied to human beings, using invasive and noninvasive procedures to evaluate endothelium-dependent vasodilatation, or the determination of plasmatic substances that indicate endothelial activation and/or damage.

 The incidence of CAD and mortality is very low in women of reproductive age but rises to a significant level in menopause women [23]. There is evidence of an association between endothelial dysfunction and reduced endogenous production of estrogens after natural or surgical menopause or premature ovarian failure in women with or without CAD [24–27]. The actions of endogenous estrogens on the cardiovascular system can be mediated directly on the vessels or indirectly through the modulation of cardiovascular risk factors, as well as on the lipid profile [28]. The direct effects of estrogen on the vascular system and which modulate the vascular tonus comprise the following 1 acute vasodilatation, increasing the synthesis and bioactivity of NO [29, 30]; 2 long-term modulation of vascular tonus, regulating the production of prostaglandins and expression of endothelial nitric oxide synthase and the endothelin gene [31]; 3 inhibition of endothelin-induced vasoconstriction [32]; and 4 inhibition of sympathetic activity [24]. In addition to these actions on the vascular tonus, estrogen exerts an antiproliferative action on the vascular smooth layer [33]. Also, it appears to have a major role in vascular remodeling, inhibiting the proliferation of the inner layer after injury [34] and increasing the expression of contractile proteins in the myocardium [35].

Disturbances in endothelial function have an important role in the physiopathology of atherosclerosis, and several lines of evidence suggest that interventions in endothelial function could modify the progress rates of atherosclerotic disease and the risk of cardiovascular events. Some studies have documented that estrogens are potent antioxidants and decrease LDL-C oxidation in vitro and in vivo [12, 13]. Studies on the mechanism of estrogen antioxidant effects have shown that estrogen strongly inhibits superoxide formation with minor effects on hydrogen peroxide and hydroxyl radical formation [14]. While estrogen decreases lipid peroxidation and formation of reactive oxygen species, [14] androgens and progestins increase oxidative stress parameters [15]. Clinical studies on humans using 17*β*-estradiol-based preparations have clearly shown decreased LDL-C oxidation, and in addition, estradiol reduces the development of early lesions of atherosclerosis, in part through the effects on lipid metabolism which reduce lipid deposits in the endothelium [36, 37].

Cevic et al. demonstrated that a high concentration of estrogen reduces the level of asymmetric dimethylarginine (ADMA), which is an endogenous competitive inhibitor of NO synthase [38]. Estradiol, by reducing ADMA, may therefore facilitate NO synthesis in endothelial cells. Hashimoto et al. [39] also demonstrated that women who had had at least 1 pregnancy followed by delivery showed a decreased level of arteriosclerosis, measured noninvasively by brachial-ankle pulse wave velocity (ba-PWV) as an indicator of arteriosclerosis. It was closely correlated with aortic arterial stiffness and the severity of atherosclerosis, compared with those who had never experienced delivery. Human umbilical vein endothelial cells exposed to high concentration of 17*β*-estradiol were used as an antiatherosclerogenic agent to demonstrate feasibility in an in vitro vascular model [40].

LOX-1, a type II membrane glycoprotein, is the major receptor for ox-LDL in endothelial cells [16]. It is also expressed by macrophages and vascular smooth muscle cells [41]. Oxidative stress and ox-LDL both alter endothelial biology by activating a specific receptor LOX-1. The activation of LOX-1 has been shown to lead to further oxidative stress in endothelial cells and the appearance of proinflammatory phenotype [17]. LOX-1 has been implicated in vascular inflammation and atherosclerotic plaque formation, progression, and destabilization [42, 43]. LOX-1, furthermore, is cleaved at the membrane-proximal extracellular domain by proteases, including a disintegrin and matrix metalloproteinases (MMPs) [18, 19] that may also be associated with plaque vulnerability or rupture, resulting in soluble LOX-1 (sLOX-1) release into the circulation [19]. In addition, plasma sLOX-1 levels were higher in males and smokers than in females and nonsmokers, probably because endogenous estrogen and smoking affect plaque vulnerability by protecting vascular cells from inflammation (the former) [8] and by inducing oxidative stress and inflammation (the latter) [44]. In experimental animal models, LOX-1 expression is closely associated with morphological plaque instability and cell apoptosis, as well as with the expression of MMPs and tissue factor, all of which are associated with plaque rupture and thrombus formation [45–47]. A study demonstrated that LOX-1 deficiency significantly decreases the formation of atherosclerotic lesions and endothelial dysfunction [48].

 It is well known that menopause or lack of endogenous estrogen is a risk factor for cardiovascular disease [49–51]. Hashimoto et al. [39] reported that women who are regularly menstruating have a decreased PWV compared with post-menopausal women of the same age and a younger age at menarche correlates with PWV reduction. This finding may support the idea that as long as women are exposed to endogenous estrogen they have decreased endothelial dysfunction. Cardiovascular risk increases after bilateral ovariectomy and in conditions associated with impaired ovarian function. Thus, ovarian dysfunction and either natural or surgical menopause have been recognized as a major risk factor for accelerated atherosclerotic vascular disease development [3, 52]. In stages of disrupted ovulatory cycling, low levels of endogenous oestrogens during premenopausal years accelerate the progression of atherosclerosis [53, 54], which can be reversed by oestrogen therapy in animals [53]. In addition, results from experimental studies and recent clinical trials indicate that oestrogen therapy started within few years after menopause, that is, before the development of severe atherosclerosis, may in fact reduce cardiovascular risk [5, 55–59]. In contrast, initiation of oestrogen therapy many years after menopause, that is, when advanced and multiple atherosclerotic lesions are present, may have no or even deleterious cardiovascular effects [5, 55–59].

In conclusion, our study demonstrated that serum sLOX-1 levels were associated with pregnancy followed by delivery which might predict endothelial dysfunction. Pregnancy followed by delivery may improve endothelial function and prevent the progress of atherosclerosis in women of reproductive age. LOX-1 can be used as a target for imaging of endothelial function. We conclude that pregnancy followed by delivery may delay the progress of arteriosclerosis and its clinical manifestations in women of reproductive age.

## Figures and Tables

**Table 1:** The clinical and demographic characteristics of study subjects.

	Group 1 ( *n* = 68)	Group 2 (*n* = 57)	*P* value
Age (years)	33.5 ± 6.1	35.5 ± 7.5	0.1
Age of menarche (years)	12.1 ± 2.3	11.9 ± 1.9	0.5
Smoke (%)	11.8%	15.8%	0.6
Family history (%)	7.4%	5.3%	0.7

*Lipid profile *(mg/dL)			
Total-C	179 ± 39	183 ± 28	0.4
LDL-C	108 ± 31	113 ± 23	0.3
HDL-C	45 ± 12	45 ± 11	0.7
Triglycerides	120 ± 66	132 ± 58	0.3

Data expressed as mean ± SD or percentage. *P* < 0.05 was accepted as statistically significant. Total-C: total cholesterol, LDL-C: low-density lipoprotein cholesterol, HDL-C: high-density lipoprotein cholesterol. Group 1: pregnancy followed by delivery group, Group 2: nongravidity group.
